# A stochastic approach to k-nearest neighbors search using a fixed radius method

**DOI:** 10.1007/s00180-025-01674-7

**Published:** 2026-01-13

**Authors:** Brahian Cano Urrego, Alexander Alsup, Jeffrey A. Thompson, Devin C. Koestler

**Affiliations:** Department of Biostatistics & Data Science, University of Kansas Medical Center, 3901 Rainbow Blvd, Kansas City, KS 66106 USA

**Keywords:** KNN search, Computational efficiency, Fixed-radius search, Branch and bound, Machine learning

## Abstract

This study aims to optimize the $\:k$-nearest neighbors search (kNN search) by reducing the computational burden of the well-known Brute-force method while providing the same solution. While there exist rule-based approaches for reducing the computational burden of the kNN search, methods that use the stochastic patterns inherent to the data are lacking. Our method leverages data structures and probabilistic assumptions to enhance the scalability of the search. By focusing on the Training set where our neighbors reside, we define a sample space that limits the $\:k$-nearest neighbors search to a smaller space. For each observation in the Query set (e.g., the set of observations for which a classification is desired), a fixed radius search is employed, with the radius stochastically linked to the desired number of neighbors. This approach allows us to find the $\:k$-nearest neighbors using only a fraction of the entire Training set in contrast to the Brute-force method, which requires distances to be calculated between each observation in the Training set and each observation in the Query set. Through simulations and a theoretical computational complexity analysis, we demonstrate that our method outperforms the Brute-force approach, particularly when the Training and Query set sample sizes are large. In addition, a benchmarked comparison of our approach and the Brute-force method on an Alzheimer’s disease data set further demonstrated this, showing a 27.57-fold improvement in total elapsed time. Overall, our stochastic approach significantly reduces the computational load of kNN search while maintaining accuracy, making it a viable alternative to traditional methods for large datasets.

## Introduction

The field of machine learning has garnered significant interest in recent years. This surge in attention is primarily due to the promise and potential of machine learning to advance and accelerate research discoveries across a broad spectrum of research areas. Supervised learning is a branch of machine learning that involves the identification of response-specific patterns in a data set with the goal of predicting or classifying future observations for which the response is unknown. Examples of supervised machine learning methods abound and include well-known methods such as: Decision Trees, Support Vector Machines, Neural Networks, and Random Forests. Among the suite of existing supervised machine learning methodologies, one of the simplest and most intuitive is $\:k$-nearest neighbors (kNN) (Fix and Hodges 1985). The kNN algorithm is a non-parametric method that uses the proximity or similarity between a query point and observations in the Training set to make classifications or predictions. It has been extensively applied across various scientific fields and adapted to address the nuances of different study designs and data types. For example, Hou et al. optimized the kNN classification by creating a KD-tree structure that includes labels of all training observations (Hou et al. 2018). Dong et al. proposed a kNN weighted imputation method to address block-missing data arising in the field of trans-omics (Dong et al. 2019). Recently, Jena et al. used a kNN filtering-based algorithm for an e-learning recommendation system (Jena et al. 2023). In addition, Kaplan et al., found kNN to be the best performing method to classify brain tumors based on magnetic resonance imaging (Kaplan et al. 2020). Despite its widespread applications and advantages, kNN suffers from computational inefficiencies, especially in the case of high-dimensionality. Importantly, this inefficiency becomes pronounced with large sample sizes in either the Query set (the set of observations for which a classification is desired) or the Training set.

Several methods have been proposed to address the computational challenges intrinsic to kNN. These methods aim to optimize the kNN search by reducing the computational burden while providing the same or an approximate solution. Specific example of such methods include: linear search (Fix and Hodges 1985), approximate nearest neighbor (Aria et al. 1998), dimensionality reduction techniques (Gates 1972; Hart 1968), and principal axis (McNames 2001). Each of these methods have specific strengths and limitations. For example, approximate nearest neighbor algorithms sacrifice exactitude for speed, while dimensionality reduction techniques might not always preserve the essential structure of the data. Despite these advancements, there remain opportunities for improvement as existing solutions do not fully address the scalability issues of kNN search, particularly in the case of extremely large datasets. Additionally, there is a need for methods that can efficiently manage high-dimensional data without significant loss of information. Furthermore, mainstream approaches rely on deterministic data structures, highlighting the potential of stochastic-based alternatives as a promising direction for research.

In this paper, we propose an approach to address the computational inefficiencies of kNN search. Our method uses a **st**ochastic approach to the k**NN** search problem using a **f**ixed-**r**adius search (STNNfr) by leveraging data structures and probabilistic assumptions to enhance the scalability of the kNN search. By leveraging the kNN search definition, we define a sample space that limits the kNN search to a smaller space. For each observation in the Query set, a fixed radius search is used where the radius is stochastically linked to the desired number of neighbors. Consequently, we require only a fraction of the Training set to find the $\:k$-nearest neighbors for a given observation in the Query set. This results in a more efficient solution for large-scale, high-dimensional data as we demonstrate both theoretically and empirically.

In what follows, we first formally introduce the kNN search problem and its most well-known algorithm, the Brute-force method. This is followed by a description of our approach, STNNfr, which seeks to address the computational shortcomings of the Brute-force method. We next describe the rationale behind our simulation scenarios and the metrics used to benchmark the approaches being compared. A computational comparison of our approach and the Brute-force method is carried out, providing an opportunity to offer guidance into hyper-parameter tuning. We finish by summarizing important insights from this work, along with a discussion of the limitations of our study and avenues for future work.

## Methods

We first review the Brute-force approach (i.e., linear search) to the $\:k$-nearest neighbor search in $\:p$-dimensions. As a means toward introducing this approach, we follow the notation of James et al. (James et al. 2013):

- $\:{\boldsymbol{X}}^{\left(q\right)}$: Query set containing $\:m$ observations (e.g., observations for which a prediction or classification is desired).
- $\:\boldsymbol{X}$: Training set. Contains the $\:n$ observations from which the $\:k$-nearest neighbors are derived.

$$ X^{{\left( q \right)}} = \left[ {\begin{array}{*{20}c} {x_{{11}}^{{\left( q \right)}} } & {\: \ldots \:} & {\:x_{{1p}}^{{\left( q \right)}} } \\ \vdots & {\: \ddots \:} & {\: \vdots } \\ {\:x_{{m1}}^{{\left( q \right)}} } & {\: \ldots \:} & {\:x_{{mp}}^{{\left( q \right)}} } \\ \end{array} } \right]_{{m \times \:p}} = \left[ {\begin{array}{*{20}c} {x_{1}^{{\left( q \right)}} } \\ {\: \vdots } \\ {\:x_{m}^{{\left( q \right)}} } \\ \end{array} } \right]\:,\:\:X = \left[ {\begin{array}{*{20}c} {x_{{11}} } & {\: \ldots \:} & {\:x_{{1p}} } \\ \vdots & {\: \ddots \:} & {\: \vdots } \\ {\:x_{{n1}} } & {\: \ldots \:} & {\:x_{{np}} } \\ \end{array} } \right]_{{n \times \:p}} \:\: = \left[ {\begin{array}{*{20}c} {x_{1} } \\ {\: \vdots } \\ {\:x_{n} } \\ \end{array} } \right] $$

- $\:{N}_{i}$ : Set of $\:k$-nearest neighbors belonging to $\:\boldsymbol{X}$ for the $\:i$-th observation in the Query set $\:{\boldsymbol{x}}_{i}^{\left(q\right)}$, $\:i=1,\dots\:,m$ based on some measure of distance or proximity.

In the standard formulation of kNN, the distance between $\:{\boldsymbol{x}}_{i}^{\left(q\right)},\:i=1,\dots\:,m\:$ and all observations in the Training set is first calculated, which we define as $\:{\boldsymbol{d}}_{i}^{\left(q\right)}=\left[{d}_{i1}^{\left(q\right)},\:{d}_{i2}^{\left(q\right)},\dots\:,\:{d}_{in}^{\left(q\right)}\right]$. Next, distances are sorted in ascending order to create the order statistics $\:{d}_{i\left(1\right)}^{\left(q\right)},{d}_{i\left(2\right)}^{\left(q\right)},\dots\:,\:{d}_{i\left(n\right)}^{\left(q\right)}$. Finally, the set $\:{N}_{i}$ is constructed to contain the $\:k$ observations in the Training set with smallest distance $\:{N}_{i}=\left\{j|\:{d}_{ij}^{\left(q\right)}\in\:\:\left\{{d}_{i\left(1\right)}^{\left(q\right)},{d}_{i\left(2\right)}^{\left(q\right)},\dots\:,{d}_{i\left(k\right)}^{\left(q\right)}\right\}\right\}$, where $\:k\le\:n$. This process is repeated for each of the $\:m$ observations in the Query set, $\:{\boldsymbol{X}}^{\left(q\right)}$.

Although the Brute-force method provides exact and reliable solutions, the computational complexity of this algorithm is $\:O\left(nm\right)$, which is not optimal. For instance, when $\:k$ is small relative to $\:n$, say 5, and $\:n$ is, for example 1000, there will be 995 observations in the Training set that will not be used for the ultimate classification or prediction of a specific query point. To curb the computational burden associated with Brute-force $\:k$-nearest neighbor search, we present a stochastic approach that uses a radius-based method with complexity no more than $\:O\left(m\left({n}^{1-\frac{1}{p}}+k\right)\right)$.

### *St*ochastic k*NN* search based on *f*ixed-*r*adius search (STNNfr)

**Fig. 1:**
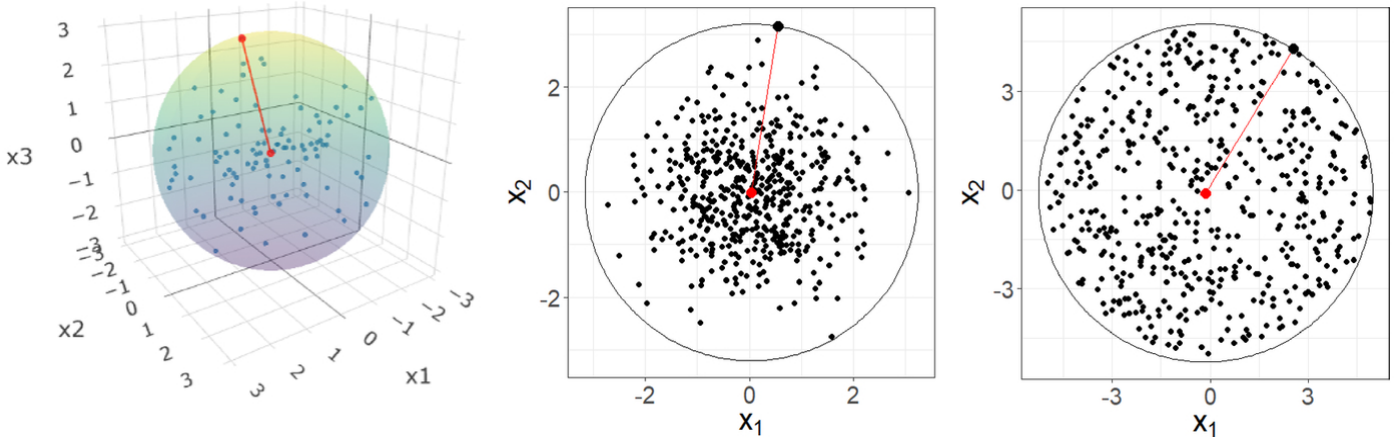
Visual representation of the construction of the Training set sample space for STNNfr. Left panel shows a 3-dimensional spherical uniform distribution, middle panel shows a 2-dimensional standard normal distribution, right panel shows a 2-dimensional uniform circular distribution

Since the search for the $\:k$-nearest neighbors of a given observation in the Query set occurs only in the Training set, we define a sample space of the Training set that limits it to a $\:p$-dimensional hypersphere that contains all observations in the Training set (Fig. 1). Subsequently, a fixed-radius search is performed for each observation in the Query set where the radius of a given point has a stochastic relationship to the desired number of neighbors. As a result, we expect to find the $\:k$-nearest neighbors for a given observation using only a fraction of the full Training set. To illustrate our approach, first define:

- $\:{\boldsymbol{x}}_{\stackrel{-}{j}}=\stackrel{-}{\boldsymbol{x}}=\left[{\stackrel{-}{x}}_{1},{\stackrel{-}{x}}_{2},\dots\:,{\stackrel{-}{x}}_{p}\right]^{\prime\:}$ as the centroid of Training set and $\:\stackrel{-}{j}$ represents its index.
- $\:{\boldsymbol{d}}_{\stackrel{-}{j}}=\left[{d}_{1\stackrel{-}{j}},{d}_{2\stackrel{-}{j}},\dots\:,{d}_{n\stackrel{-}{j}}\right]^{\prime\:}$ is a vector of distances between each of the Training set observations and the overall centroid of the Training set, where $\:d_{{j\mathop j\limits^{ - } }} = \left\| {x_{j} - \bar{x}_{j} } \right\| = \left\| {x_{j} - \bar{x}} \right\|\:\:for\:j = 1,\: \ldots \:,\:n$ in the case of the Euclidean distance metric.
- $\:r=\mathrm{max}\left(\left\{{d}_{1\stackrel{-}{j}},{d}_{2\stackrel{-}{j}},\dots\:,{d}_{n\stackrel{-}{j}}\right\}\right)\:\:$as the maximum distance calculated between all observations in the Training set to the centroid of the Training set.

Next, we create a $\:p$-dimensional hypersphere $\:S$ of radius $\:r$ centered at $\:\stackrel{-}{\boldsymbol{x}}$ such that $\:S$ contains all the observations in the Training set (Fig. 1). In doing so, the kNN search is reduced to $\:S\:\subseteq\:{\mathbb{R}}^{p}$. An advantage of this framework is that we know that by construction, no neighbors can be found outside $\:S$.

To find the $\:k$-nearest neighbors of $\:{\boldsymbol{x}}_{i}^{\left(q\right)}$, create $\:p$-dimensional hypersphere $\:{S}_{i}$ with unknown radius $\:{r}_{i}$, centered at $\:{\boldsymbol{x}}_{i}^{\left(q\right)}$.

The novelty here is to solve for $\:{r}_{i}$ such that at least $\:k$-nearest neighbors fall inside the hypersphere $\:{S}_{i}$. In order to do this, assume that the Training data $\:\boldsymbol{X}$ originates from a spherical uniform distribution, with probability distribution function, $\:g\left(\boldsymbol{x}|r,\boldsymbol{z}\right)=\frac{1}{\mathrm{v}\mathrm{o}\mathrm{l}\mathrm{u}\mathrm{m}\mathrm{e}\left(S\right)}=\frac{1}{{\pi\:}^{\frac{p}{2}}\:\frac{{\mathrm{r}}^{p}}{{\Gamma\:}\left(\frac{p}{2}+1\right)}}$ for $\:\left\{ {x:\left\| {x - z} \right\| \le \:r} \right\}$, where $\:{\Gamma\:}\left(a\right)$ is the Gamma function and $\:\boldsymbol{z}$ is the centroid of the hypersphere. We refer interested readers to (Sun and Chen 2008) for further details regarding the spherical uniform distribution. Next, we seek to find an $\:{r}_{i}$ such that:

$$\:P\left( {S_{i} \:} \right) = \tau \:,\:\:\mathrm{where}\:S_{i} = \left\{ {x:\left\| {x - x_{i}^{{\left( q \right)}} } \right\| \le \:r_{i} } \right\} $$

Here, $\:\tau\:\in\:\left(\mathrm{0,1}\right]$ is a pre-specified probability threshold and $\:{\boldsymbol{x}}_{i}^{\left(q\right)}$ is the centroid of $\:{S}_{i}$. For example, finding $\:{r}_{i}$ such that $\:P\left({S}_{i}\:\right)=\tau\:=0.1$ would imply that $\:{S}_{i}$ contains 10% of the observation in the Training set in probability; this would be the case if $\:k$ is set to 10 ($\:k\:=\:10$) and the Training set contains 100 observations ($\:n\:=\:100$).

By the definition of our sample space and assumption that the Training set follows a spherical uniform distribution, we have:

$$\:P\left( {S_{i} \:} \right) = P(S_{i} \: \cap \:S) = \frac{{volume(S_{i} \: \cap \:S)\:}}{{volume\left( S \right)}} $$

In the above expression, volume $\mathrm{(}{S}_{i}\:{\cap}\:S\mathrm{)}$ can be calculated using existing derivations and adapted formulas for calculating the intersection of two $\:p$-dimensional hyper-spheres (Li 2010; User 2013). This enables us to proceed with the following:

Define $$\:{d}_{i\stackrel{-}{j}}^{\left(q\right)}=\Vert\:{\boldsymbol{x}}_{i}^{\left(q\right)}-\stackrel{-}{\boldsymbol{x}}\Vert\:$$ and

$$ \:volume\left( {S_{i} \: \cap \:S} \right) = \left\{ {\begin{array}{*{20}c} {\:if\:\:d_{{i\mathop j\limits^{ - } }}^{{\left( q \right)}} \ge \:r_{i} + r\:,\:\:\:\:\:\:\:\:\:\:\:\:\:\:\:\:\:\:\:\:\:\:\:\:\:0\:\:\:\:\:\:\:\:\:\:\:\:\:\:\:\:\:\:\:\:\:\:\:\:\:\:\:\:\:} \\ {\:if\:\:d_{{i\mathop j\limits^{ - } }}^{{\left( q \right)}} \le \:\:\:\left| {r_{i} - r} \right|,\:\:\:\:\:\:\:\pi \:^{{\frac{p}{2}}} \:\frac{{\min \left( {r_{i} \:,r} \right)^{p} }}{{\Gamma \:\left( {\frac{p}{2} + 1} \right)}}\:\:\:\:\:\:\:\:\:\:\:\:\:\:\:\:\:} \\ {\:else,\:\:\:\:\:\:\:\:\:\:\:\:\:\:\:\:\:\:\:\:\:\:\:\:\:\:\:\:\:\:\:\:V_{p}^{{cap}} \left( {r,c_{1} } \right) + V_{p}^{{cap}} (r_{i} ,c_{2} )} \\ \end{array} } \right. $$

where,

$$\:{c}_{1}=\frac{{{d}_{i\stackrel{-}{j}}^{\left(q\right)}}^{2}+{r}^{2}-{r}_{i}^{2}}{2{d}_{i\stackrel{-}{j}}^{\left(q\right)}},\:{\:\:\:\:\:\:\:\:\:\:\:\:c}_{2}=\frac{{{d}_{i\stackrel{-}{j}}^{\left(q\right)}}^{2}-{r}^{2}+{r}_{i}^{2}}{2{d}_{i\stackrel{-}{j}}^{\left(q\right)}}$$

and

$$\:{V}_{p}^{cap}\left(r^{\prime\:},a\right)=\left\{\begin{array}{c}if\:a\ge\:0,\:\:\:\:\frac{{\pi\:}^{\frac{p}{2}}}{2}\:\frac{{{r}^{{\prime\:}}}^{p}}{{\Gamma\:}\left(\frac{p}{2}+1\right)}{I}_{1-{a}^{2}/{{r}^{{\prime\:}}}^{2}}\left(\frac{p+1}{2},\frac{1}{2}\right)\:\\\:if\:a<0,\:\:{\pi\:}^{\frac{p}{2}}\frac{{{r}^{{\prime\:}}}^{p}}{{\Gamma\:}\left(\frac{p}{2}+1\right)}-{V}_{p}^{cap}\left({r}^{{\prime\:}},-a\right)\end{array}\right.$$.

such that $\:{I}_{1-\alpha\:}\left(b,c\right)\:$is the regularized incomplete beta function. Next, we numerically find the value of $\:{r}_{i}$. Defining $\:f\left({r}_{i}\right|r,p,{d}_{i\stackrel{-}{j}}^{\left(q\right)},\tau\:)=P\left({S}_{i}\:\right)-\tau\:$, it suffices to find the roots of $\:f$

1 $$\:f\left( {r_{i} |r,p,d_{{i\mathop j\limits^{ - } }}^{{\left( q \right)}} ,\tau \:} \right) = 0 \Leftrightarrow P\left( {S_{i} \:} \right) - \tau \: = 0 \Leftrightarrow\:\frac{{volume(S_{i} \:\cap\:S)}}{{volume(S)}} - \tau \: \Leftrightarrow \frac{{volume(S_{i} \:\cap\:S)}}{{\pi \:^{{\frac{p}{2}}} \:\frac{{r^{p} }}{{\Gamma \:\left( {\frac{p}{2} + 1} \right)}}}} - \tau \: = 0 $$

Once $\:{r}_{i}$ is obtained, we use a fixed-radius search algorithm. In our case, we use a KD-tree based algorithm (Bentley 1975), in which we partition the data using the Training set. Note that the partition only needs to be created once. Subsequently, we submit a query of radius $\:{r}_{i}$ and if less than $\:k$-nearest neighbors are found, $\:\tau\:$ is increased. We repeat the above steps until $\:{k}^{{\prime\:}}$ neighbors have been found, where $\:k\le\:{k}^{{\prime\:}}\le\:n$.

Lastly, we compute the sorted distances to create the order statistics $\:{d}_{i\left(1\right)},{d}_{i\left(2\right)},\dots\:,\:{d}_{i\left({k}^{{\prime\:}}\right)}$, and construct $\:{N}_{i}$ to consist of $\:k$ observations in the Training set with smallest distance. Specifically:

$$\:{N}_{i}=\left\{j|\:{d}_{ij}\in\:\:\left\{{d}_{i\left(1\right)},{d}_{i\left(2\right)},\dots\:,{d}_{i\left(k\right)}\right\}\right\}$$.

**Fig. 2:**
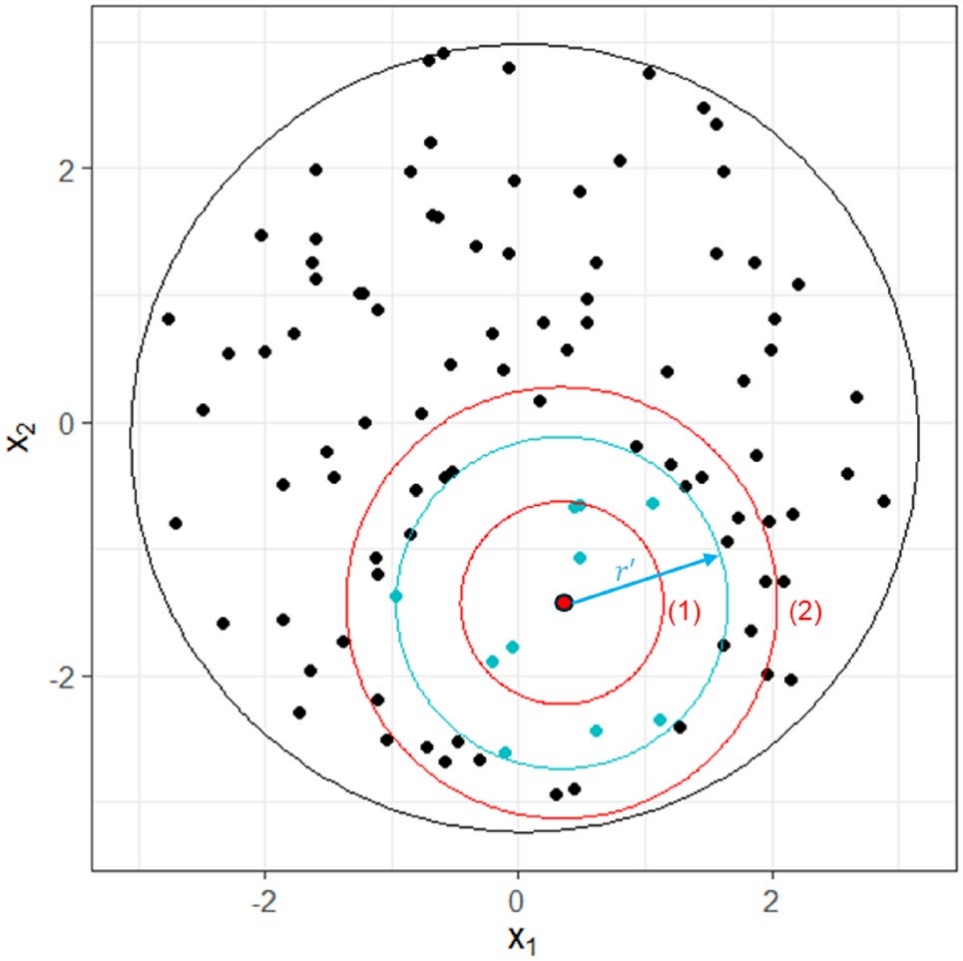
Example of the iterative process to find the $\:k$-nearest neighbors. For a query point (red dot) $\:r{\prime\:}$ is the radius of the unknown hypersphere (blue circle) determined by the *k*-th nearest neighbor. The algorithm iterates twice (red (1) and red (2)) and stops once a circle bigger than the unknown circle (blue circle) is found. This circle (red circle $\:\left(2\right)$) will contain the *k*-nearest neighbors

It is worth mentioning that every query point has its own unknown hypersphere that contains the $\:k$-nearest neighbors, where its radius $\:r^{\prime\:}$ is equal to the distance between the query point and the $\:k$-th nearest neighbor (Fig. 2, blue circle/dots). This hypersphere has an unknown probability $\:\tau^{\prime\:}$, where $\:{0<\tau\:}^{{\prime\:}}\le\:1$. To guarantee that our method captures the $\:k$-nearest neighbors, we require a hypersphere whose volume is equal to or greater than unknown hypersphere (Fig. 2, red circle $\:\left(2\right)$). Since we sweep $\:\tau\:$ from some initial value up to 1.0, this approach will eventually create a hypersphere that contains the $\:k$-nearest neighbors.

### Optimization based on ring groups

One caveat of our approach concerns the apparent need to solve Eq. (1) as many times as there are observations in the Query set. However, further inspection of Eq. (1) reveals that it does not depend on the actual coordinates of the observations in the Query set but rather: (1) their distance $\:{d}_{i\stackrel{-}{j}}^{\left(q\right)}$ to the overall centroid of the Training set, (2) the radius $\:r$, (3) the desired threshold $\:\tau\:$, and (4) the number of dimensions/features $\:p$. Thus, for fixed values of $\:r,\:\tau\:,\:\mathrm{a}\mathrm{n}\mathrm{d}\:p$, observations in the Query set with the same distance $\:{d}_{i\stackrel{-}{j}}^{\left(q\right)}={d}_{{i}^{{\prime\:}}\stackrel{-}{j}}^{\left(q\right)},\:\:i\ne\:{i}^{{\prime\:}}$, would result in the same $\:{r}_{i}$. Consequently, we propose reducing the number of equations by grouping the observations in the Query set homogeneously in terms of their distance $\:{d}_{i\stackrel{-}{j}}^{\left(q\right)}$. Let us define $\:G$ as the number of ring groups such that:

$$ \:group\left( {x_{i}^{{\left( q \right)}} } \right) = \left\{ {\begin{array}{*{20}c} {1,\:\:\:\:\:\:\:\:if\:\:\delta \:_{0} = 0 \le \:d_{{i\mathop j\limits^{ - } }}^{{\left( q \right)}} \le \:\delta \:_{1} \:\:\:\:\:\:\:\:} \\ {\:2,\:\:\:\:\:\:\:\:if\:\:\:\delta \:_{1} < d_{{i\mathop j\limits^{ - } }}^{{\left( q \right)}} \le \:\delta \:_{2} \:\:\:\:\:\:\:\:\:\:\:\:\:\:\:} \\ {\: \vdots } \\ {\:G,\:\:\:\:\:\:\:if\:\:\:\delta \:_{{G - 1}} < d_{{i\mathop j\limits^{ - } }}^{{\left( q \right)}} \le \:\delta \:_{G} \:\:\:\:\:\:\:\:\:} \\ {\:G + 1,\:\:\:if\:\:\:d_{{i\mathop j\limits^{ - } }}^{{\left( q \right)}}> \delta \:_{G} = r\:\:\:\:\:\:\:\:\:\:\:\:\:\:\:\:\:\:\:} \\ \end{array} } \right. $$

where $\:{\delta\:}_{g}$ is the radius of a sub-hypersphere with centroid $\:{S}_{\stackrel{-}{j}}$ having probability $\:g/G$. More specifically:

$$ \:P\left( {S_{{\mathop j\limits^{ - } }} } \right) = \frac{g}{G}\: \Leftrightarrow \:P\left( {S_{{\mathop j\limits^{ - } }} \cap \:S} \right) = \frac{{volume(S_{{\mathop j\limits^{ - } }} \: \cap \:S)}}{{volume(S)}} = \frac{g}{G}\: \Leftrightarrow \:\frac{{volume(S_{{\mathop j\limits^{ - } }} )}}{{volume(S)}} = \frac{g}{G}\: $$

$$\:\iff\:\frac{{\pi\:}^{\frac{p}{2}}\:\frac{{{\delta\:}_{g}}^{p}}{{\Gamma\:}\left(\frac{p}{2}+1\right)}}{{\pi\:}^{\frac{p}{2}}\:\frac{{\mathrm{r}}^{p}}{{\Gamma\:}\left(\frac{p}{2}+1\right)}}=\frac{g}{G}\iff\:\frac{{{\delta\:}_{g}}^{p}}{{\mathrm{r}}^{p}}=\frac{g}{G}\:\iff\:\:\:{\delta\:}_{g}=r\:\:\sqrt[p]{\frac{g}{G}}\:,\:\:\:g=1,\:\dots\:,\:G.$$

**Fig. 3:**
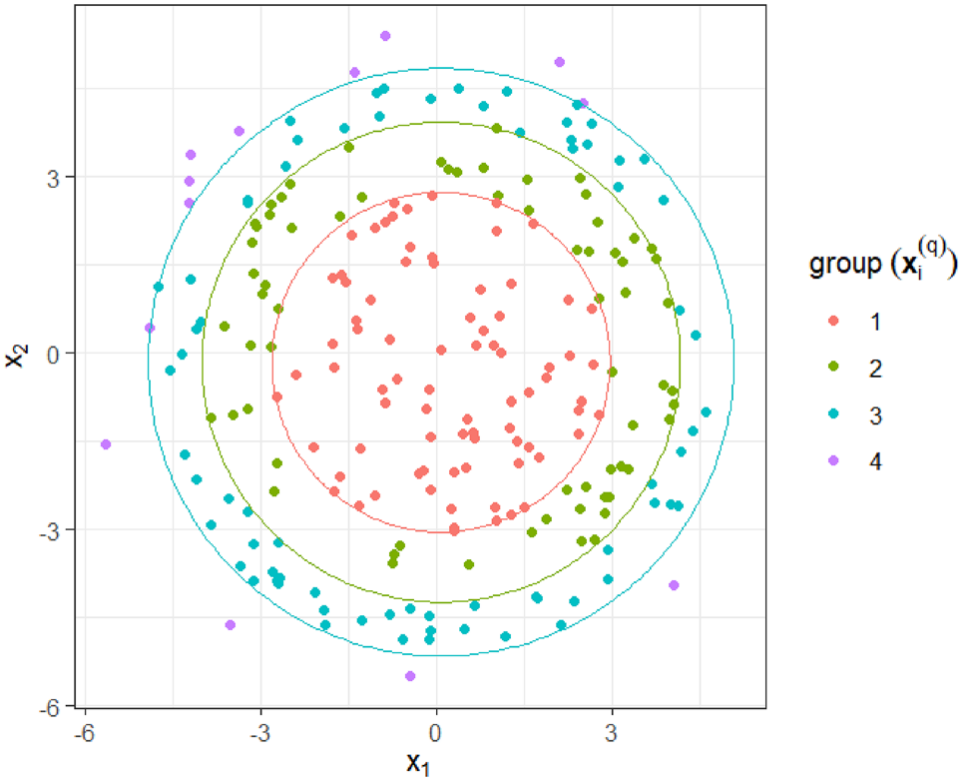
A 2-Dimensional representation of the ring grouping where $\:G=3$

This grouping is exemplified in (Fig. 3).

Once each $\:{\boldsymbol{x}}_{i}^{\left(q\right)}$ has been classified, we proceed to establish a common equation to determine the fixed radius search of all observations belonging to the group, solving numerically $\:f\left({r}_{i,g}|r,p,{d}_{i\stackrel{-}{j}}^{\left(q\right)}={\delta\:}_{g},\tau\:\right),\:\mathrm{f}\mathrm{o}\mathrm{r}\:g=1,\dots\:,G$, where $\:{r}_{i,g}$ is the new radius for all the observations in Query set that belong to group $\:g$. Note, the main difference when solving this equation is that we use $\:{d}_{i\stackrel{-}{j}}^{\left(q\right)}={\delta\:}_{g}$, which is the outer part of the ring. This reduces the number of equations from $\:m$ to $\:G$, where $\:G<m$. This variant of our search algorithm has a computational complexity of $\:O\left(m\left({n}^{1-\frac{1}{p}}+k\right)\right)$. Finally, we follow the same steps outlined Sect. 2.1, with the exception that $\:{r}_{i}\:$ is replaced by $\:\:{r}_{i,g}$, for $\:i=1,\:\dots\:,\:m.$ The full pipeline of the proposed STNNfr algorithm is summarized in Table 1. Taken together, we note that for $\:n\ge\:1$ we have that:

$$\:O\left(\:{mn}^{1-\frac{1}{p}}+mk\right)=O\left(m\left({n}^{1-\frac{1}{p}}+k\right)\right)\le\:O\left(m\left({n}^{1-\frac{1}{p}}+n\right)\right)<O\left(m\left(n+n\right)\right)=$$

$$\:O\left(m\left(2n\right)\right)=O\left(mn\right)\Rightarrow\:O\left(\:{mn}^{1-\frac{1}{p}}+mk\right)<O\left(mn\right)$$

Thus, the proposed STNNfr algorithm is theoretically more efficient than the Brute-force kNN search method.

**Table 1:** Pipeline and complexity of stochastic kNN fixed radius search (STNNfr)

Step	Computational Cost
1.Calculate the centroid of the training set	$\:n$
2.Distances from training points to centroid	$\:n$
3.Defining the rings groups	$\:G$
4.Assigning groups to the query set	$\:m$
5.Solving $\:G\times\:t$ numerical equations	$\:G\times\:\:t\times\:\mathrm{O}\left(1\right)$
6.Create searching structure (KD-tree)	$\:pnlog\left(n\right)$
7.Iterative radius search	$\:m\times\:t\times\:\left({n}^{1-\frac{1}{p}}+k\right)$
8.Return Neighbors	$\:\mathrm{mlog}\left({k}^{{\prime\:}}\right),\:{k}^{{\prime\:}}=c\times\:\:n$ Worst case$\:\to\:m\mathrm{log}\left(n\right)$
Total complexity	$\:O\left(\:{mn}^{1-\frac{1}{p}}+mk\right)$

## Simulation study

Even though the complexity of STNNfr has been derived theoretically and shown to be superior to the Brute-force method (Eq. 2), for two algorithms with identical complexity, one can be faster than the other due to simplifications associated with Big O notation. On the other hand, algorithms are not only evaluated based on their speed but also their memory consumption. For these reason, the goals of our simulation study were two-fold: (1) compare the computational time, total elapsed time, and the memory consumption of STNNfr relative to the standard Brute-force search method across different simulated different sets and (2) shed light on the selection/specification of the hyper parameter, $\:G$.

For our simulation studies, we generated data for both the Query and Training sets from: a multivariate standard normal distribution; a multivariate t-distribution with 10 degrees of freedom and an identity covariance matrix; and a spherical uniform distribution with a radius of 1.0. The purpose of generating data from a multivariate normal and t-distribution was to assess the performance of STNNfr when the distributional assumptions of the Training set observations are violated. The choice of 10 degrees of freedom for multivariate t-distribution is motivated by the desire to generate a more scattered set data and heavier density around the outer parts of the sphere. We considered samples sizes of 100, 1000, 10,000, and 100,000 for the Training set and samples sizes of 50, 100, and 200, in the Query set, with the additional constraint that $\:m\le\:n$. The number of features/dimensions, $\:p$, assumed four values: 20, 50, 100, and 250. The number of nearest neighbors $\:k$ was proportional to $\:n$: $\:1,\:0.16n,\:0.33n,\:$and $\:0.5n$, rounded to nearest whole number. The number of ring groups $\:G$ was set to: 1, 2, 5, and 10. Each combination of parameter settings was replicated a total of 25 times to account for random variation.

In an effort to minimize the number of iterations needed by STNNfr with respect to set of prespecified thresholds $\:\: T=\left({\tau\:}^{\left(1\right)},\dots\:,\:{\tau\:}^{\left(t\right)}\right)$, we started the search with $\:{\tau\:}^{\left(1\right)}\:$equal to proportion of nearest neighbors with respect to the Training set sample size $\:k/n$. We considered ten values in total, $\:t=10$, which ranged from $\:k/n\:$ to $\:1,$ evenly spaced. We preserved this structure for $\:T$ across all simulation scenarios. Thus, under the worst-case scenario, the algorithm would iterate a total of $\:t=10$ times. Moreover, for the sake of simplicity and without loss of generality we used the well-known and widely used Euclidean distance to calculate proximity/similarity between observations.

For each combination of parameters, we report: (1) the $\:k$-nearest neighbors of all observations in the Query set; (2) the CPU and elapsed time averaged across the 25 replications; (3), the memory allocation averaged across the 25 replications; and (4) specific to STNNfr, the average number of iterations required for the algorithm averaged across the $\:m$ observations in the Query set and averaged again across the 25 replications.

Due to the computational complexity of the simulations considered, simulations were conducted on the University of Kansas High-Performance Computing (HPC) cluster. The R statistical programming language (https://cran.r-project.org/) was used to carry out the simulations (R Core Team 2023). The implementation of the Brute-force method was based on the algorithm described in Sect. 2.1. In contrast to loops (e.g., for loops, while loops, etc.), we used vectorized functions to improve the efficiency of our implementation. The implementation of STNNfr included functions from MASS (Venables and Ripley 2002) and dbscan R packages (Hahsler et al. 2019). CPU and elapsed were calculated using the *system.time()* function in R, and memory allocation was calculated using the *mem_change()* function from pryr R package (Wickham 2023).

The R code implementing the methods and simulations is available on the following GitHub repository: bencuben/2025_STNNfr: Code and material for the manuscript.

## Results

### Simulation results

For all simulation scenarios considered and for each replication, the $\:k$-nearest neighbors of the $\:m$ observations in the Query set were exactly the same for both the Brute-force method and STNNfr, and in the same order. This observation serves to demonstrate the consistency in results between STNNfr and the standard, Brute-force search method.

STNNfr can be considered as a branch and bound algorithm, where we divide the problem into several iterations. Our expectation is that by selecting a “good set” of hyperparameters, the average number of iterations are as close to 1 as possible. Our simulation results confirmed that regardless of the parameter settings, the number of iterations required until convergency was, on average, equal to 1.

**Fig. 4:**
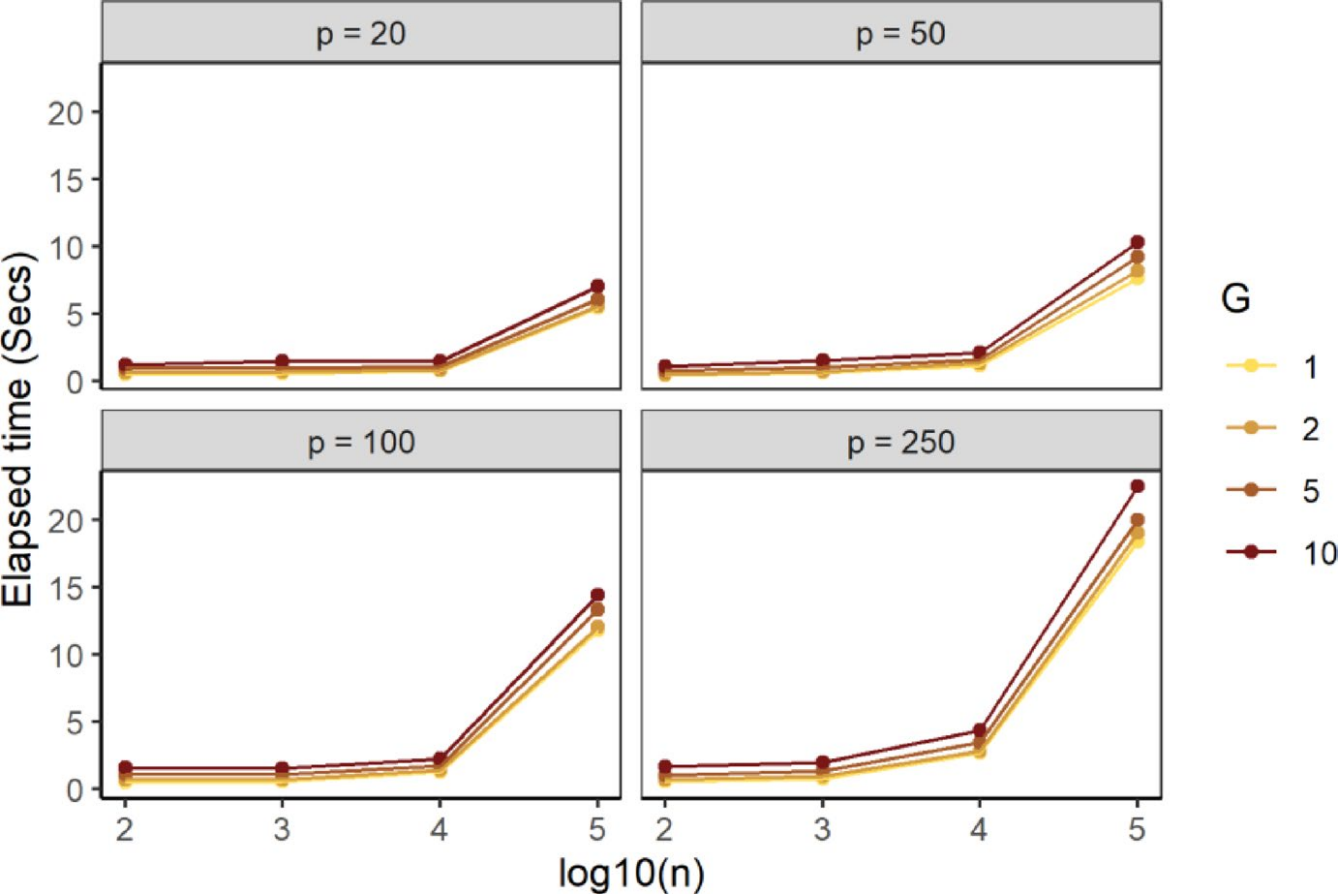
Average elapsed time of STNNfr within the same simulation scenario as a function of the Training set sample size in log10 scale (x-axis), number of ring groups (color), the dimension of the feature-space (panels). Data were generated from the spherical uniform distribution

As shown in Fig. 4, smaller values of $\:G$ result in a smaller elapsed time, suggesting that an overall radius for all observations could be a suitable strategy. Although larger values of $\:G$ were not considered in the simulation, we expect that elapsed time would increase as $\:G$ increases. This aligns with the rationale in Sect. 2.2, where the algorithm was simplified by solving fewer numerical equations instead of having to solve numerical equations for each observation in the Query set ($\:G=m$). It is worth mentioning that the selection of $\:G$ does not have any impact on the accuracy of the kNN search; it only impacts the running time.

**Fig. 5:**
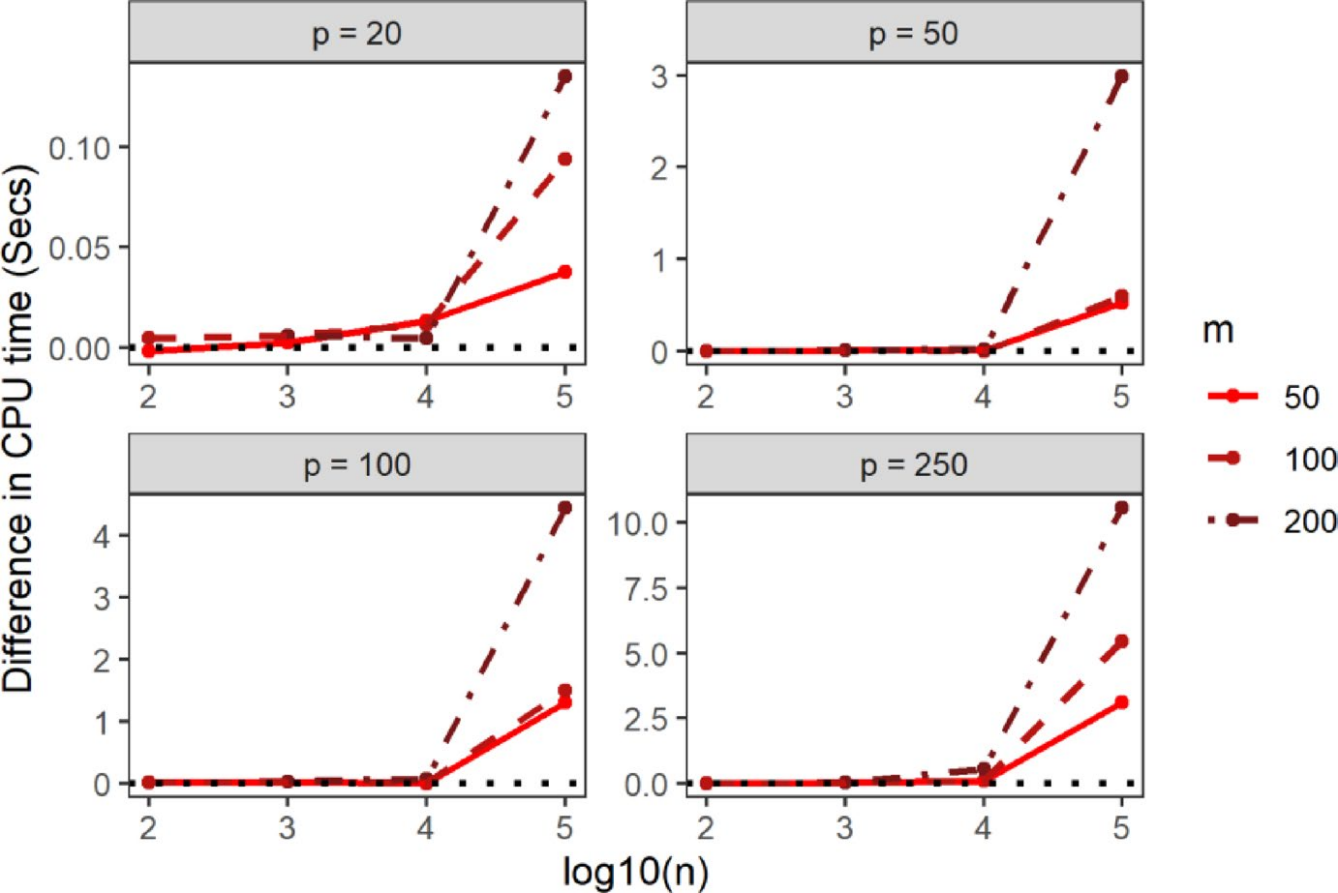
Average difference in CPU time (y-axis) of the Brute-force and STNNfr methods (Brute-force minus STNNfr) within same simulation scenario as a function of the Training set sample size on the log10 scale (x-axis), Query set sample size (color), the dimension of the feature-space (panels). Data were generated from a spherical uniform distribution with the number of ring groups assumed to be one, $\:G=1$

**Fig. 6:**
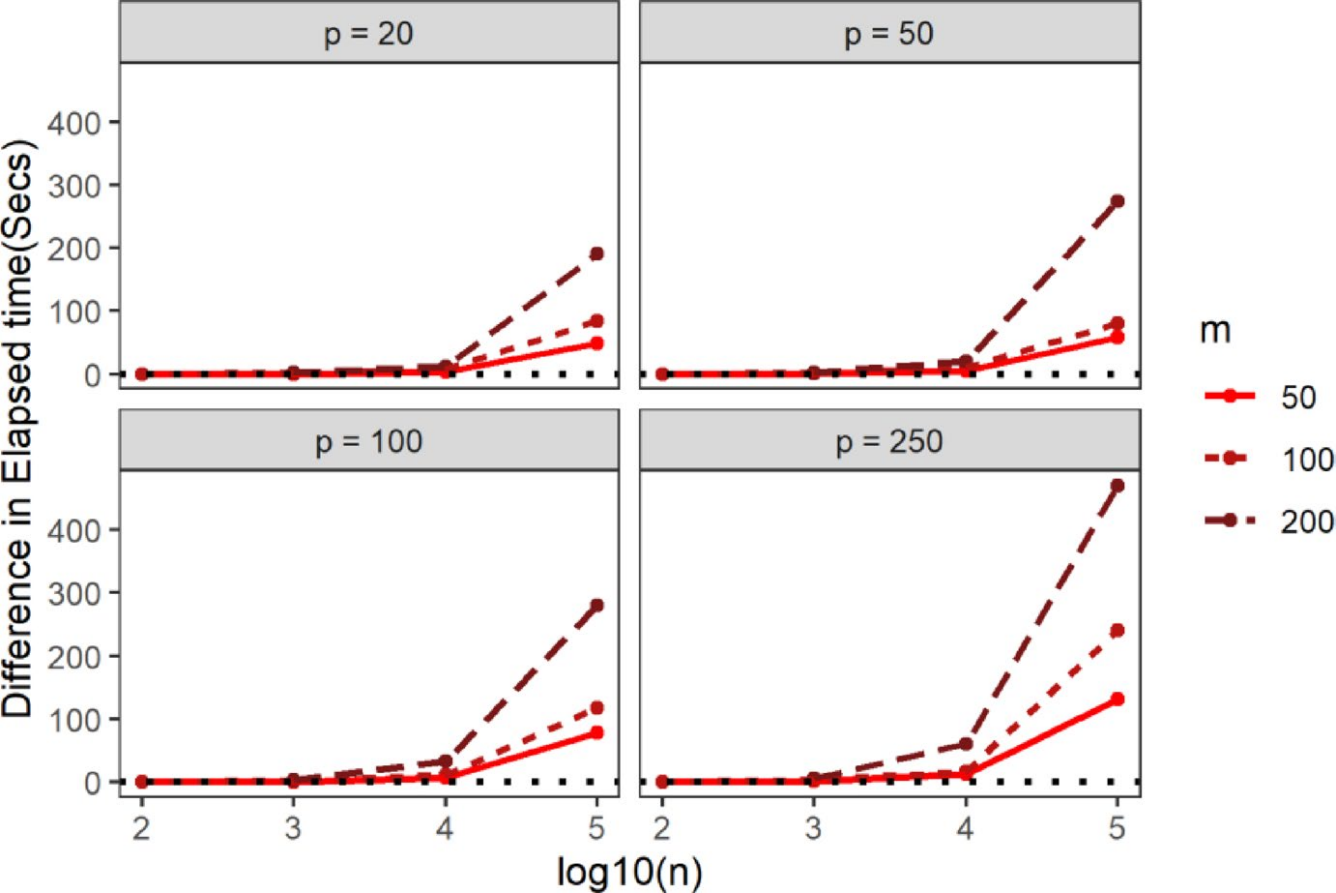
Average difference in elapsed time (y-axis) between the Brute-force method and STNNfr (Brute-force minus STNNfr) within same simulation scenario as a function of the Training set sample size on the log10 scale (x-axis), Query set sample size (color), and the dimension of the feature-space (panels). Data were generated from a spherical uniform distribution with the number of ring groups assumed to be one, $\:G=1$

**Fig. 7:**
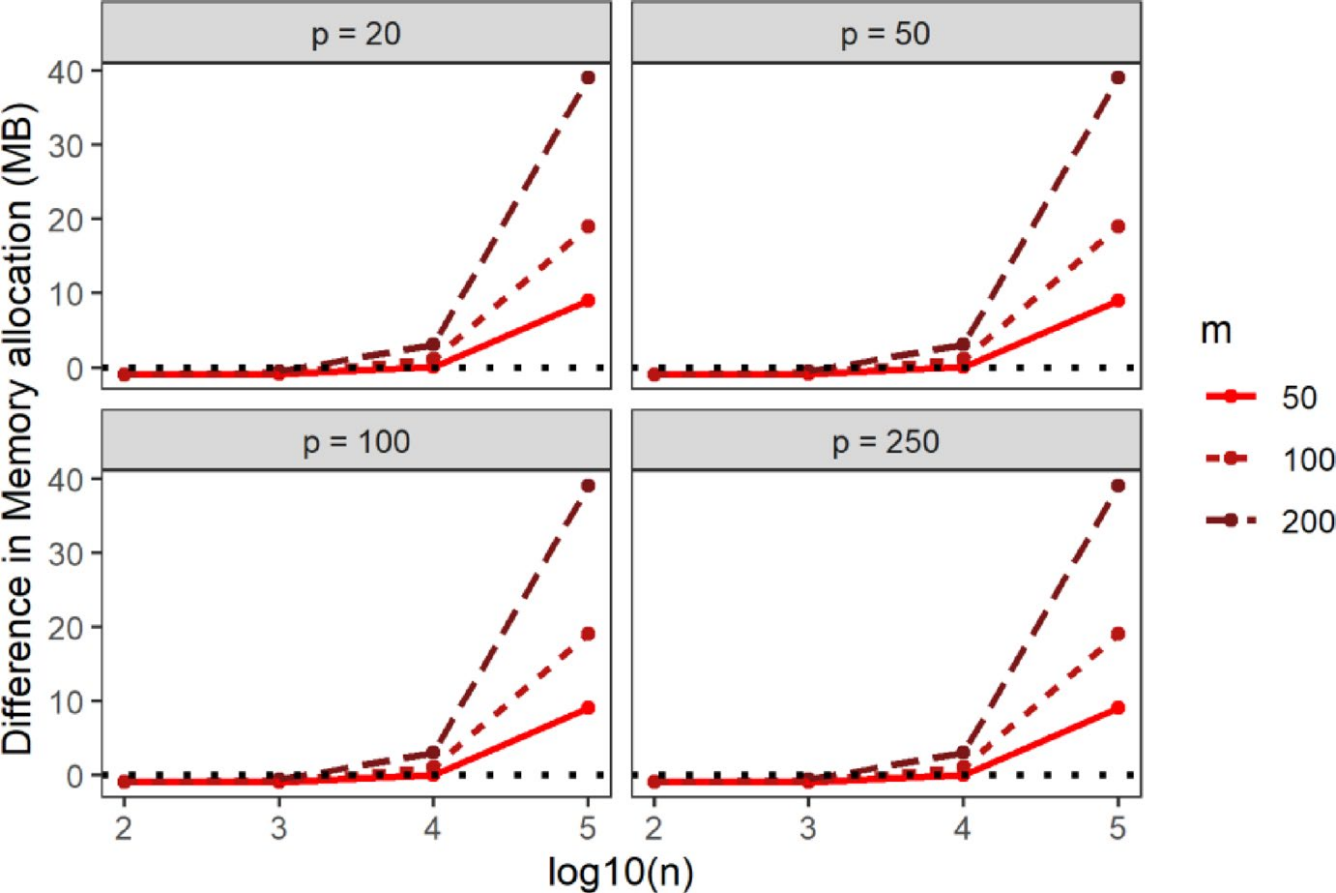
Average difference in memory allocation (y-axis) between the Brute-force method and STNNfr (Brute-force minus STNNfr) within same simulation scenario as a function of the Training set sample size on the log10 scale (x-axis), Query set sample size (color), and the dimension of the feature-space (panels). Data were generated from a spherical uniform distribution with the number of ring groups assumed to be one, $\:G=1$

Next, to benchmark STNNfr’s computational efficiency against the Brute-force method, we compared the difference in CPU time, elapsed time, and memory allocation under identical simulation parameters and setting $\:G=1$. In Figs. 5, 6 and 7, values above 0 indicate better performance for STNNfr. Generally, as the number of dimensions, Training set sample size, or Query set sample size increased, the average CPU time, elapsed time, and memory allocation difference between STNNfr and Brute-force increased. While STNNfr performed slightly worse for small dimensions ($\:n=100,\:\:p=20,\:50$), it demonstrated significant improvements in high-dimensional cases. Specifically, Fig. 6 highlights an average of an ~ 500-second improvement in elapsed time when $\:n=100,\:000,\:\:p=250,\:m=200$. These findings align with our theoretical derivation in Eq. (2), confirming STNNfr’s reduced computational complexity.

**Fig. 8:**
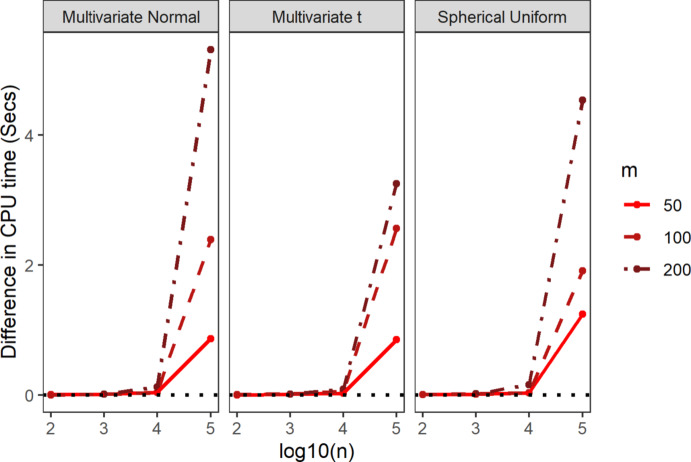
Average difference in CPU time (y-axis) between the Brute-force method and STNNfr (Brute-force minus STNNfr) as a function of Training set sample size on the log10 scale (x-axis), Query set sample size (color), and underlying distribution used to generate the data (panels)

Lastly, to evaluate the performance of STNNfr under distributional violation, data were generated from both a multivariate normal and multivariate t-distribution (df = 10). Figure 8 (Appendix) presents the average difference in CPU time between the Brute-force method and STNNfr as a function of the Training and Query set sample sizes when data are generated from a spherical uniform, multivariate normal, and multivariate t-distribution. The results in Fig. 8 demonstrate that irrespective of the distribution used to generate the data, STNNfr consistently outperforms Brute-force method in high-dimensional settings. Notably, while STNNfr exhibits a general advantage across distributions relative to the Brute-force method, its performance gains are slightly less pronounced under the multivariate t-distribution.

### Application for alzheimer’s disease dataset

To further benchmark STNNfr to the Brute-force approach, we considered the dataset Diagnosis AlzheimeR WIth haNdwriting (DARWIN) (Cilia et al. 2022) available at: DARWIN - UCI Machine Learning Repository. DARWIN includes data collected on 88 Alzheimer’s patients and 85 healthy participants. Each study participant was assigned 25 tasks that assessed the participant’s capability to perform in four different categories: graphics, copy, memory, and dictation. For each task, 18 different metrics were recorded from a tablet equipped with a pen that allowed the researchers to track the space-coordinates of the pen at every moment. As a result, 450 features were collected.

Since the number of patients was relatively small, we generated synthetic observations from healthy participants and Alzheimer’s patients. We first estimated the mean vectors and variance-covariance matrices for both groups. Assuming normal distributions, we then sampled 50,000 synthetic observations from each population using their respective mean vectors and variance-covariance matrices. Next, data was split into Training and Query sets using 90/10 allocation while maintaining a consistent ratio of healthy to Alzheimer’s patients. This resulted in $\:n=90000,\:m=10000,\:\:p=450$. For the sake of numerical stability and tackling the effect of the scale in measurements, the Training set was preprocessed by normalizing it (subtracting the mean and dividing by the standard deviation) and the same preprocessing for the Query set was used. Finally, for the kNN search we set $\:k=15$ and $\:G=1$.

The Brute-Force method obtained a CPU time of 179 s, an elapsed time of 49,329 s and a memory usage of 8.65 MB. In comparison, STNNfr achieved a CPU time of 20 s (8.95-fold improvement in efficiency); an elapsed time of 1,789 s (27.57-fold improvement in efficiency); and a memory usage of 8.98 MB (3.67% larger memory consumption). These results confirm the insights from simulations and theoretical calculations, demonstrating that STNNfr is much faster than the Brute-force method, with the trade-off of slightly increased memory usage in high-dimensional situations.

## Discussion

In this paper we introduced a novel method to find the $\:k$-nearest neighbors based on a stochastic approach to fixed-radius search, providing an alternative strategy to the kNN search problem. This problem is of high relevance for scientific and applied fields, where challenges of classification, regression, and unsupervised clustering of “big-data” require computational efficiency (e.g., health claims data, EHR, national repositories, etc.). Our primary aim was to compare the computational efficiency of STNNfr and the well-known Brute-force method. As reported, STNNfr consistently matched the Brute-force method in identifying the $\:k$-nearest neighbors for all scenarios and replications, demonstrating its reliability and consistency in the solutions arrived at via the Brute-force method. STNNfr averaged 1 iteration irrespective of the parameter settings. For the scenarios considered, $\:G=1$ provided the best performance across the simulations. In practice, there might be cases in which a different selection of $\:G$ would be optimal; nevertheless, the only consequence of mis specifying $\:G$ is an increase in convergence time. Computational efficiency benchmarks in both our simulation study and computational complexity calculations showed STNNfr to outperform the Brute-force method, especially for high-dimensional situations. Specifically, the application in the DARWIN data set revealed a 23-fold improvement in in elapsed time. However, for small dimensions, STNNfr was slightly slower compared to the Brute-force method, indicating areas for coding-based improvement.

Despite the noted contributions of our method and its performance relative to the Brute-force approach, no study is free from limitations. Our simulation study evaluated STNNfr against the Brute-force method, that, although it is not widely used in practice, serves as a fundamental benchmark for kNN search due to its exhaustive nature and neutrality. It imposes no partitioning or distribution assumptions, ensuring a fair evaluation of efficiency gains achieved by STNNfr. Consequently, future comparisons against more commonly used methods, such as KD-tree and Ball-tree, present an opportunity to further contextualize STNNfr’s effectiveness.

Distributional assumptions must be carefully evaluated when imposed. While our findings indicate that STNNfr exhibits robustness to distributional violations, there may be applications where such violations pose challenges. The choice of a spherical uniform distribution was motivated by the need to establish a worst-case scenario, in which all training points are evenly dispersed.

Another potential limitation of our study is that we fixed our simulations to $\:m\le\:n$ to limit the amount of memory needed to conduct our simulation study and since we believe that in many applications, the size of the Query set tends to be smaller than the Training set. Nevertheless, based on the results presented we expect that STNNfr would still outperform the Brute-force method when $\:m>n$. It is also important to note that to enable a fair comparison between STNNfr and the Brute-force method, we used our own coded version of the Brute-force method since several of the existing implementations are coded in the general purpose programming language, C++ (ISO/IEC 14882:2023, 2023). By coding the Brute-force method in R we sought to avoid a situation where differences between the two approaches were driven by the level where functions are running by standardizing their coding language. There are some current inefficiencies in the coded STNNfr algorithm that are opportunities for future work. These include redundant computation and memory optimization. Lastly, the current implementation of the STNNfr is based on KD-tree, however other more efficient alternatives could be explored such as fixed-radius NN search based on sorting (SNN) (Chen and Güttel 2024).

Additional future work includes: expanding the simulation scenarios where needed; implementation of some parts of our algorithm in C++; inclusion of competitors coded in C++, like KD-tree, Ball-tree, and Brute-force. We also plan to analyze the impact of data transformations on our algorithm’s performance and results, along with expanding to different assumed distributions for the Training and Query set beyond the multivariate standard normal, the multivariate t, and spherical uniform distributions that were considered herein. Future work should also be directed toward innovating a more methodical approach to define $\:T$, and alternatives to define the initial value of $\:\tau\:$ in the STNNfr algorithm. We also plan to analyze the performance of STNNfr using different distance metrics other than Euclidean distance, however, we expect STNNfr to exhibit superior performance as compared to the Brute-force method on the basis of our theoretical calculations of its complexity and the invariance of our calculations relative to the chosen distance metric.

In conclusion, we introduced a new method to find the $\:k$-nearest neighbors based on a stochastic approach to fixed radius search (STNNfr). To our knowledge, STNNfr is the first method to address the kNN search problem from a stochastic perspective. We compared the computational efficiency of STNNfr to the well-known Brute-force method using both simulations studies and theoretical computational complexity calculations. Our results demonstrate favorable performance of STNNfr, especially for large Training and Query sample sizes. We see STNNfr having an impact in areas such as biomedicine, economics, and social sciences, by helping to reduce the computational burden of finding similar/proximal observations.

## Appendix

See Fig. 8 below.
